# Association Between Human Papillomavirus Vaccination and the Risk of Hashimoto’s Thyroiditis: A Cross-Sectional Study

**DOI:** 10.3390/vaccines13050490

**Published:** 2025-04-30

**Authors:** Yifan Yin, Liang Ye, Min Chen, Hao Liu, Jingkun Miao

**Affiliations:** 1 Department of Pediatrics, Chongqing Health Center for Women and Children, Chongqing 401120, China; 115205@hospital.cqmu.edu.cn (Y.Y.); yeallen2020@163.com (L.Y.); cm19921115@gmail.com (M.C.); liuhao-1983@live.com (H.L.); 2 Department of Pediatrics, Women and Children’s Hospital of Chongqing Medical University, Chongqing 401120, China

**Keywords:** human papillomavirus, vaccine, Hashimoto’s thyroiditis, thyroid peroxidase antibody, thyroglobulin antibody, autoimmune diseases

## Abstract

**Background/Objectives**: Concerns about the occurrence of autoimmune diseases are one of the main reasons influencing the uptake of the human papillomavirus (HPV) vaccine. Limited evidence exists regarding the relationship between HPV vaccination and the risk of Hashimoto’s thyroiditis (HT). Therefore, the purpose of this study was to examine the association between HPV vaccination and the risk of HT development in American women. **Methods**: Using the National Health and Nutrition Examination Survey (NHANES) data from 2007 to 2012, we conducted a cross-sectional study of 2717 women aged 18–59 with comprehensive data on relevant HPV vaccination status, HPV DNA vaginal swab results, and thyroid function. The relationship between HPV vaccination and the risk of HT development was explored by weighted logistic regression, while the association between HPV vaccination and thyroid peroxidase antibodies (TPOAb)/thyroglobulin antibodies (TGAb) levels was analyzed by weighted linear regression. **Results**: In the fully adjusted model, HPV vaccination was associated with an 87% decrease in the risk of developing HT (OR 0.13; 95% CI 0.02, 0.76). Furthermore, weighted linear regression demonstrated significant negative associations between HPV vaccination and TPOAb levels (−22.27 (−34.86, −9.68), *p* = 0.001) and TGAb levels (−7.53 (−14.88, −0.18), *p* = 0.045). HPV vaccination was significantly negatively correlated with the risk of HT development and TPOAb/TGAb levels. **Conclusions**: We advocate for adherence to vaccination guidelines, which could confer dual protective benefits against HPV and potentially reduce the risk of HT development.

## 1. Introduction

The human papillomavirus (HPV) stands as a prevalent pathogen globally, typically transmitted through sexual contact, with initial exposure often coinciding with the onset of sexual activity. HPV’s enduring presence within the human host holds the potential to incite malignancies in the penile, vulvar, vaginal, and anal regions, along with specific oropharyngeal carcinomas and anogenital warts [1]. Vaccines designed to preemptively combat HPV demonstrate proven efficacy and safety [2], yet the uptake of such vaccines remains suboptimal, trailing the adoption rates of other recommended adolescent immunizations in the U.S. [3]. Refusal of the HPV vaccine is attributed to several key factors: a shortfall in medical endorsements; a perceived non-necessity of immunization; a general lack of awareness, coupled with apprehensions regarding its safety and potential adverse reactions from caregivers [4,5]. The association between large-scale vaccination and autoimmune diseases is a subject of controversy in both Europe and North America [6,7]. In previous studies, there have been reports of adverse drug reactions associated with HPV vaccines, with some cases involving autoimmune disease [8]. However, some studies suggest that there is no association between HPV vaccines and autoimmune diseases [9,10,11]. To alleviate public concerns and enhance HPV vaccination rates, further research on the relationship between HPV vaccines and autoimmune diseases is necessary.

Hashimoto’s thyroiditis (HT), recognized as one of the most prevalent autoimmune disorders, is the leading cause of hypothyroidism [12]. The characteristics of HT include thyroid enlargement, lymphocytic infiltration of thyroid tissue, and the presence of specific antithyroid antibodies, such as thyroid peroxidase antibodies (TPOAb) and thyroglobulin antibodies (TgAb). Elevated levels of anti-thyroid peroxidase (anti-TPO) antibodies are widely regarded as a high-risk factor for HT and are considered to have strong predictive value. Although anti-thyroglobulin (anti-Tg) antibodies are also associated with the disease, their independent predictive ability is relatively limited. In clinical practice, testing for anti-TPO antibodies is useful for the early identification and management of patients with HT [13,14]. Moreover, the global incidence of HT has surged notably in recent decades [15], from 27 per 100,000 to 448 per 100,000 [16], with a particularly marked spike among women—rates are eight times higher than in men [17]. Although significant progress has been made in understanding the pathogenesis of HT over the last few decades, the exact mechanisms and optimal treatments for HT remain unclear.

Previous studies have reported multiple cases of HT following COVID-19 and influenza vaccination [18,19,20]. These findings suggest the need for attention to potential vaccine-induced thyroid autoimmune diseases. Current safety studies on HPV vaccines focus on overall safety assessments, and so far, no evidence has been found linking HPV vaccination to autoimmune diseases [21,22,23]. However, these cohort studies have certain limitations: (1) they predominantly enroll women under 26 years of age; (2) they lack detailed questionnaires, resulting in insufficient covariate data for multivariate analyses; and (3) they focus on comprehensive safety assessments without in-depth research on specific diseases.

Our study utilized data from the 2007–2012 National Health and Nutrition Examination Survey (NHANES), an authoritative source that captures a comprehensive national representation of vaccination status and detailed thyroid function metrics among U.S. residents. Scientific sampling design, wide sample coverage, detailed questionnaires, and standardized experimental testing procedures make these data still representative. Considering that the primary recipients of the HPV vaccine and the susceptible population for HT are both females, our study primarily investigates the association between HPV vaccination and HT among women. If the incidence of HT is higher in women who received HPV vaccination in this large cross-sectional study, the association between HPV vaccination and the risk of HT needs to be further investigated.

## 2. Materials and Methods

### 2.1. Study Population

NHANES is a cross-sectional, biannual survey conducted by the Centers for Disease Control and Prevention designed to gauge the nutrition and health profiles of the American populace. Methodologies for both population and sample surveys are thoroughly documented on the NHANES website (https://www.cdc.gov/nchs/nhanes/?CDC_AAref_Val=https://www.cdc.gov/nchs/nhanes/index.htm, accessed on date 31 January 2025).

**Figure 1:**
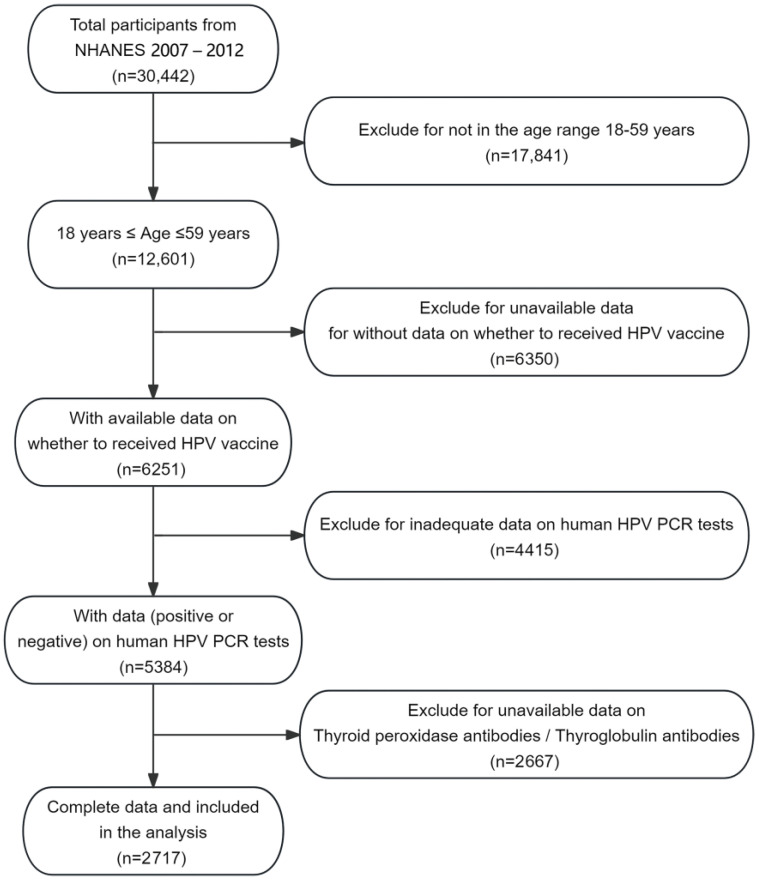
Flow chart for selecting participants from NHANES 2007–2012.

Our inquiry specifically extracted a subsection of the NHANES database extending from 2007 to 2012, incorporating three two-year data collection cycles, chosen for their encompassing thyroid function data. This investigation hones in on female respondents between the ages of 18 and 59—the demographic pertinent to HPV vaccine administration and the availability of Human Papillomavirus Polymerase Chain Reaction (HPV PCR) test data. Consequently, the study’s analytical parameters were confined to examining females within this age bracket, ensuring a focus on relevant HPV vaccination records, PCR test outcomes, and thyroid antibody prevalence. We established exclusion criteria to refine the dataset: individuals outside the 18–59 age range (*n* = 8879), missing HPV vaccination status (*n* = 6350), and absent thyroid antibody data (TPOAb and TGAb) (*n* = 2667). After applying these criteria, the final sample size encompassed 2717 participants from an initial pool of 30,442, in adherence to the study’s inclusion standards (Figure 1).

### 2.2. Thyroglobulin Assay

The Access thyroglobulin (Tg) assay is a simultaneous one-step “sandwich” assay. A sample is added to a reaction vessel, along with a biotinylated mixture of four monoclonal anti-Tg antibodies, streptavidin-coated paramagnetic particles, and a monoclonal anti-Tg antibody–alkaline phosphatase conjugate. The biotinylated antibodies and the serum or plasma Tg bind to the solid phase, while the conjugate antibody reacts with a different antigenic site on the thyroglobulin molecule. After incubation, materials bound to the solid phase are held in a magnetic field, while unbound materials are washed away. The chemiluminescent substrate Lumi-Phos™ 530 (Lumigen, Inc., Southfield, MI, USA) is then added to the reaction vessel, and the light generated is measured with a luminometer. The light production is directly proportional to the concentration of Tg in the sample, as determined from a stored multi-point calibration curve.

### 2.3. Thyroglobulin Antibody Assay

The thyroglobulin antibody (TGAb) detection assay employs a sequential dual-phase immunoenzymatic approach following the sandwich principle. Initially, the specimen is introduced into a vessel containing thyroglobulin-coated paramagnetic particles. Following primary incubation, a magnetic field retains particle-bound components while allowing elimination of unbound substances through washing. Subsequently, alkaline phosphatase-conjugated thyroglobulin is introduced, which selectively attaches to captured TGAb molecules present in the original sample. After secondary incubation and thorough washing to eliminate residual unbound conjugate, the addition of the chemiluminescent reagent Lumi-Phos™ 530 generates a light signal quantifiable via luminometry. The resulting luminescence exhibits direct proportionality to the specimen’s TGAb concentration, with quantification achieved through interpolation against a pre-established multi-point reference calibration.

### 2.4. Thyroid-Stimulating Hormone Assay

The determination of human thyroid-stimulating hormone (hTSH) utilizes a third-generation dual-site “sandwich” immunoenzymatic methodology via the Access HYPERsensitive platform. The analytical process begins with sample introduction into a reaction chamber containing multiple components: paramagnetic particles coated with mouse monoclonal anti-hTSH antibodies (immobilized through goat anti-mouse antibodies), goat anti-hTSH–alkaline phosphatase conjugate, and buffered protein matrix. During incubation, hTSH molecules simultaneously bind to the solid-phase immobilized monoclonal antibodies and react with the enzyme-labeled goat antibodies at distinct antigenic epitopes. Magnetic separation subsequently retains the bound immunocomplexes, while unbound elements are eliminated through washing procedures. Addition of the Lumi-Phos™ 530 chemiluminescent substrate generates light emission quantified by luminometric measurement. The luminescence intensity exhibits direct proportionality to the specimen’s hTSH concentration, which is calculated through reference to a previously established multi-point calibration function.

### 2.5. Free T4 Assay

The two-step enzyme immunoassay for Access Free T4 (FRT4) begins with the addition of biotin-coupled monoclonal anti-Thyroxine (T4) antibodies, sample, protein buffer, and streptavidin-coated solid phase to the reaction vessel. Initial incubation facilitates binding between the biotinylated antibodies, solid phase, and free thyroxine (T4) molecules present in the specimen. A magnetic separation technique then retains bound materials while eliminating unbound components through washing. The process continues with the introduction of buffered protein solution containing triiodothyronine (T3)-alkaline phosphatase conjugate, which attaches to unoccupied anti-T4 antibody sites. Following secondary incubation and another magnetic separation/washing cycle, the addition of Lumi-Phos™ 530 chemiluminescent substrate generates measurable light inversely correlating with free T4 concentration. Quantification relies on interpolation against a pre-established multi-point calibration curve.

### 2.6. Human Papillomavirus DNA Testing

Vaginal swab specimens underwent DNA extraction for Human Papillomavirus (HPV) detection via the Roche Linear Array HPV Genotyping Test. The methodology employs consensus polymerase chain reaction (PCR) amplification targeting the L1 region using biotinylated PGMY09/11 primers, with concurrent β-globin amplification serving as internal quality control.

The primer mixture facilitates simultaneous detection of genital HPV variants alongside human β-globin sequences. Post-amplification, PCR products undergo hybridization with immobilized oligonucleotide probes detecting 37 distinct HPV genotypes (comprising types 6, 11, 16, 18, 26, 31, 33, 35, 39, 40, 42, 45, 51, 52, 53, 54, 55, 56, 58, 59, 61, 62, 64, 66, 67, 68, 69, 70, 71, 72, 73, 81, 82, 83, 84, 89, and IS39) plus β-globin. Colorimetric visualization enables genotype determination through reference template comparison.

Specimens yielding negative results for both HPV and β-globin indicate insufficient DNA quantity/quality and are deemed unsuitable for evaluation. Positive Linear Array results provide molecular evidence of HPV infection.

### 2.7. Variables

In our analysis, we defined a TPOAb level more than 9.0 IU/mL or a TGAb level more than 115.0 IU/m as a high-risk factor for HT and used the risk of HT as the outcome variable.

HPV vaccination serves as the primary predictor variable, and the data are collected from the immunization section of the questionnaire. Respondents affirming receipt of the vaccine constituted the ‘vaccinated’ cohort, whereas negative responses identified the ‘unvaccinated’ group, omitting participants who were uncertain or chose not to disclose their vaccination status. Records with missing data were also discarded. The immunization questionnaire section provided HPV vaccination information, categorized by administration frequency (single dose, double dose, or complete three-dose regimen).

Data acquisition encompassed multiple domains via standardized assessment instruments. Demographic profiling included age, sex, race, marital circumstances, socioeconomic indicators (family poverty income ratio), and educational history. Behavioral assessment documented alcohol intake patterns and smoking status. Clinical evaluation recorded cardiovascular conditions (hypertension), lipid abnormalities (hypercholesterolemia), and metabolic disorders (diabetes). Pharmacological documentation focused on medications for blood pressure and glucose management. Qualified healthcare personnel conducted physical examinations and laboratory assessments at mobile assessment facilities. Race was categorized as Mexican American, non-Hispanic Asian, non-Hispanic White, non-Hispanic Black, and other. Education stratification employed four tiers: college or above, some college, high school or equivalent, and less than high school. Tobacco exposure classification established two groups based on lifetime cigarette consumption thresholds: smokers (≥100 cigarettes) and non-smokers (<100 cigarettes).

### 2.8. Statistical Analysis

The analysis adhered to NHANES’s survey methodology and analytical protocols. Continuous variables were articulated through medians and interquartile ranges when skewed and means with standard deviations when normally distributed. Categorical variables were summarized by percentages. The chi-square and Fisher’s exact tests facilitated categorical comparisons, while continuous attributes were examined via Mann–Whitney U tests or independent *t*-tests, as appropriate.

Statistical methods included multivariate logistic regression—deployed to estimate the odds ratio (OR) linking HPV vaccination with HT—and multivariate linear regression to scrutinize interrelationships between HPV vaccination and thyroid antibody levels. Model 1 offered an unadjusted baseline, while Model 2 extended to include age and race/ethnicity. Model 3 further incorporated an extensive array of adjustments, sweeping in sociodemographic elements, lifestyle factors, and pre-existing medical conditions.

We also conducted subgroup analyses across the variables to discern any effect modification, adding another dimension to our understanding of the data.

Powered by R software version 4.3.1 (http://www.R-project.org) combined with EmpowerStats (http://www.empowerstats.com, X&Y Solutions, Inc., Boston, MA, USA), the analytical process deemed results with a two-tailed *p*-value of less than 0.05 as bearing statistical significance.

### 2.9. Ethics Approval and Consent to Participate

Ethical sanction for the NHANES survey protocol comes from the Ethics Review Committee of the National Center for Health Statistics (NCHS). All subjects partaking in the survey provided informed consent in writing (https://www.cdc.gov/nchs/nhanes/about/erb.html?CDC_AAref_Val=https://www.cdc.gov/nchs/nhanes/irba98.htm, accessed on date 31 January 2025). It is important to highlight that the analysis was predicated on data that are publicly available; thus, no separate ethical clearance was obligatory for our examination.

## 3. Results

### 3.1. Characteristics of Participants

Table 1 displays the clinical and laboratory characteristics of the subjects. The study included 2667 participants based on NHANES 2007–2012, with 164 individuals receiving the HPV vaccine. Over half of the population was Non-Hispanic White (67%) and had a higher education level reaching high school above (65%). Compared to participants who did not receive the HPV vaccine, those who were vaccinated with the HPV vaccine were more likely to be younger (median age 22), have a high school education or higher, be unmarried, have normal blood pressure and lipid levels, a BMI within the normal range (25–30 kg/m^2^), no diabetes, be non-smokers, have low income, test negative for TPO antibodies, and be free from HPV infection.

Details regarding HPV vaccination status are presented in Table 2. Among the 2717 participants, 164 had received at least one dose of the HPV vaccine, yielding a vaccination rate of 6.04%. Of those vaccinated, 76.22% received the vaccine prior to age 26, while only 3.05% underwent vaccination after age 45. Additionally, 47.10% of vaccinated females completed the full three-dose regimen.

### 3.2. The Relationship Between HPV Vaccination and the Risk of HT Development

Multivariate logistic regression analyses were employed to examine the association between HPV vaccination and the risk of HT (Table 3). Across multiple models, we consistently observed an inverse relationship. In the crude Model 1, HPV vaccination was significantly linked to a decline in HT risk (OR 0.21; 95% CI 0.07, 0.58; *p* < 0.05). Model 2, which controlled for sociodemographic factors such as age and race, also supported a protective effect of HPV vaccination, as indicated by a 72% decreased risk for HT (OR 0.28; 95% CI 0.10, 0.78; *p* < 0.05). The fully adjusted Model 3 reaffirmed this association, showing an 87% reduction in the likelihood of developing HT among the vaccinated individuals (OR 0.13; 95% CI 0.02, 0.76; *p* < 0.05) (Table 3). Independent logistic regression analysis of HPV infection against the risk of HT development yielded no compelling correlation (Table A1).

The association between HPV vaccine dose and the risk of HT was further analyzed (Table 4). Across multiple statistical models, receiving either one or three doses of the vaccine was associated with a lower risk of developing HT compared to unvaccinated individuals. In crude model 1, completing the three-dose regimen was significantly linked to a reduced HT risk (OR 0.099; 95% CI 0.018, 0.535; *p* < 0.05). After adjusting for sociodemographic factors, including age and race, this association remained significant (OR 0.136; 95% CI 0.026, 0.725; *p* < 0.05). In the fully adjusted Model 3, the protective effect of completing three vaccine doses was reconfirmed (OR 9.93 × 10^−7^; 95% CI 1.19 × 10^−7^, 8.32 × 10^−6^; *p* < 0.001) (Table 4).

### 3.3. The Relationship Between HPV Vaccination and TPOAb/TGAb

Elevated levels of TPOAb and/or TGAb are recognized risk factors for HT. Through multivariate linear regression, we investigated the relationship between HPV vaccination status and thyroid antibody levels (Table 5). Participants with HPV vaccination had lower TPOAb and TGAb levels. Aligning with results from the logistic analyses, a significant negative correlation was found between HPV vaccination and TPOAb levels (−22.27 IU/mL [95% CI −34.86, −9.68]; *p* = 0.001) in Model 3, and a similar trend was uncovered for TGAb levels (−7.53 IU/mL [95% CI −14.88, −0.18]; *p* = 0.045). Our results indicate a significant negative correlation between HPV vaccination and TPOAb/TGAb levels. Participants who received the HPV vaccine may exhibit lower levels of TPOAb/TGAb.

We further explored the association between vaccination dose and thyroid autoantibody levels (Table 6). In the fully adjusted model, completion of three vaccine doses was significantly associated with lower TPOAb levels (−26.72 IU/mL [95% CI −37.80, −15.64]; *p* < 0.001), and a similar reduction was observed for TGAb levels (−7.24 IU/mL [95% CI −14.36, −0.11]; *p* = 0.047). The results showed that completing the three-dose HPV vaccination regimen was significantly correlated with reduced TPOAb/TGAb levels.

### 3.4. Subgroups Analysis Between HPV Vaccination and the Risk of HT Development

**Figure 2:**
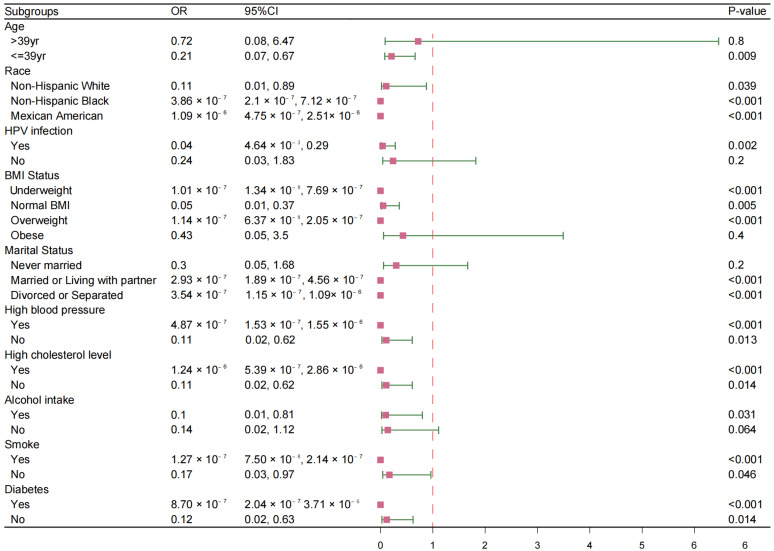
Subgroups analysis between HPV vaccination and the risk of Hashimoto’s thyroiditis. OR: odd ratios, CI: confidence interval.

To dissect these findings further, subgroup analyses were conducted (Figure 2), stratified by various factors, including age, race, marital status, BMI, alcohol consumption, diabetes, smoking status, hypertension, hyperlipidemia, and HPV infection status. The negative correlation between HPV vaccination and HT persisted across most subgroups, including smokers; those with hypertension, hyperlipidemia, or diabetes; and among various racial groups including non-Hispanic Whites, non-Hispanic Blacks, and Mexican Americans. However, no significant correlations were observed for certain subgroups, specifically, those over the age of 39 (*p* = 0.8), individuals without HPV infection (*p* = 0.2), persons categorized as obese (*p* = 0.4), those never married (*p* = 0.2), and non-drinkers (*p* = 0.064).

### 3.5. Subgroups Analysis Between HPV Vaccination and TPOAb/TGAb

**Figure 3:**
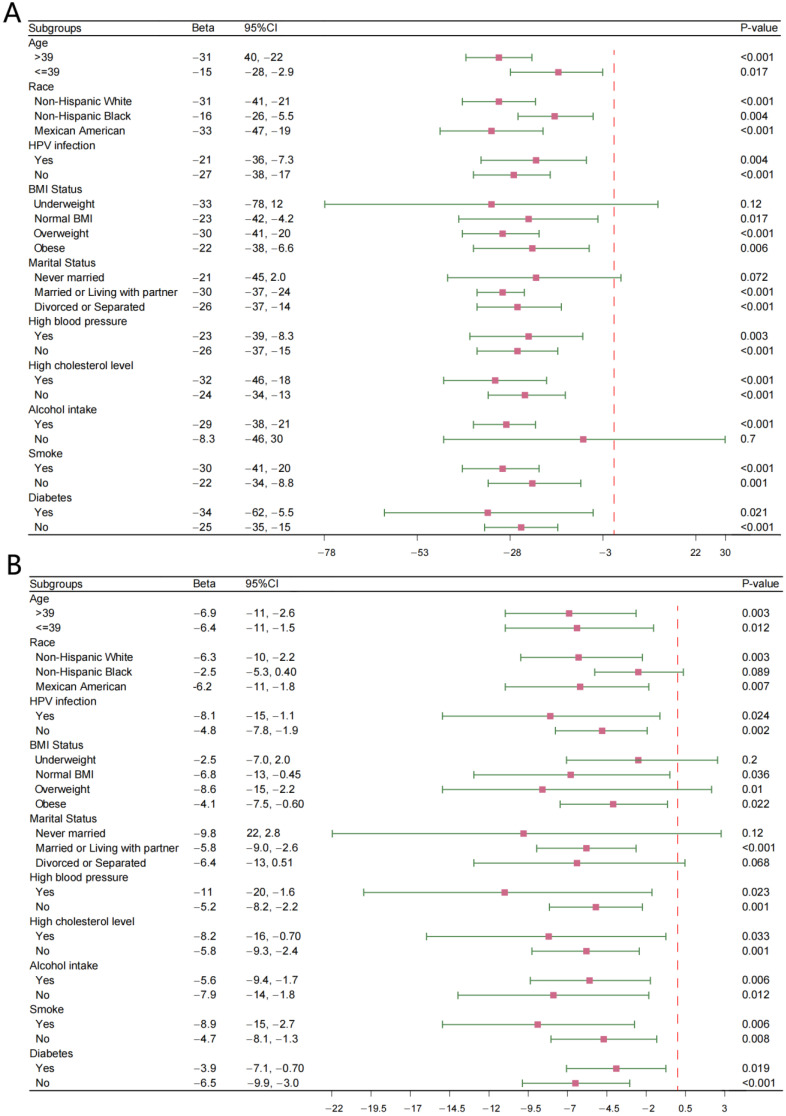
(**A**) Subgroups analysis between HPV vaccination and thyroid peroxidase antibody; (**B**) Subgroups analysis between HPV vaccination and thyroglobulin antibody. Beta: standard regression coefficient; CI: confidence interval.

In the subgroup analysis of TPOAb levels, the selected subgroups are consistent with those used in the previous subgroup analyses, including age, race, HPV infection, high blood pressure, high cholesterol level, smoking, and diabetes (Figure 3A). In all subgroups of age, race, HPV infection, high blood pressure, high cholesterol level, smoking, and diabetes, HPV vaccination is significantly negatively correlated with TPOAb levels. However, in subgroups such as underweight (*p* = 0.12), never married (*p* = 0.12), and no alcohol intake (*p* = 0.7), there is no significant negative correlation between HPV vaccination and TPOAb levels.

Regarding the subgroup analysis of TGAb levels, in all subgroups of age, HPV infection, high blood pressure, high cholesterol level, alcohol intake, smoking, and diabetes, HPV vaccination is significantly negatively correlated with TGAb levels (Figure 3B). However, in subgroups such as non-Hispanic Black (*p* = 0.089), underweight (*p* = 0.2), never married (*p* = 0.12), and divorced or separated (*p* = 0.068), there is no significant negative correlation between HPV vaccination and TGAb levels.

## 4. Discussion

Recognized worldwide as the most common sexually transmitted infection, HPV has been targeted for prevention through vaccination initiatives since 2006 [24]. In the United States, the quadrivalent HPV vaccine, which confers protection against HPV types 16, 18, 6, and 11, received approval for administration in females between the ages of 9 and 26 year [25]. Extensive research has demonstrated the vaccine’s efficacy and cost-effectiveness in thwarting both HPV-related diseases and their precursor [26,27]. Despite these positive findings, apprehensions concerning the vaccine’s safety remain a significant public health dialogue [28], underscored by anecdotal evidence linking HPV vaccination to autoimmune disorders [29,30,31].

HT is the predominant chronic autoimmune ailment impairing thyroid functionality, commonly leading to hypothyroidism. Diagnosis currently hinges on clinical insights, ultrasonography, and serological markers of thyroid autoimmunity, notably TPOAb and TGAb antibodies, which serve as the most effective diagnostic criteria [17]. Notably, the prevalence of HT skews significantly towards the female demographic [17], yet inquiries into potential links between HPV vaccination in females and the risk of HT have been scant, thus stirring public concern regarding the safety of the vaccine, especially in relation to thyroid autoimmunity.

Our cross-sectional analysis showed that 76.22% of women received the HPV vaccine before the age of 26. Among those vaccinated, 47.10% completed the three-dose regimen. We observed a significant inverse relationship between HPV vaccination and HT risk, with vaccinated individuals showing an 87% reduction in HT risk (OR 0.13; 95% CI 0.02, 0.76). Additionally, completing all three doses of the vaccine significantly lowered the risk of HT (OR 9.93 × 10^−7^; 95% CI 1.19 × 10^−7^, 8.32 × 10^−6^; *p* < 0.001). Subgroup analysis further suggested that HPV vaccination may reduce HT risk in women aged 39 years or younger. This age group falls well within the recommended vaccination bracket, which advocates for initial immunization at 11–12 years, with the possibility for early vaccination at 9 years, and catch-up vaccinations for those aged 13 to 26 years who have not yet received the vaccine [32,33]. Therefore, receiving the HPV vaccine within the recommended age range may help prevent the occurrence of HT. In the “Married or Living with a partner/Divorced or Separated” population, there is a significant negative correlation between HPV vaccination and the risk of HT. Despite no significant association between HPV vaccine and the risk of HT among the “Never married” women, we still recommend HPV vaccine before marriage. Receiving the HPV vaccine before marriage not only helps prevent the occurrence of HPV infection but may also be significantly negatively correlated with HT after marriage. No significant negative correlation was observed between abstaining from alcohol and HT. In the population that abstains from alcohol, there is no significant negative correlation between HPV vaccination and HT. While the impact of lifestyle factors such as smoking and alcohol consumption on HT etiology remains debated, emerging evidence suggests that moderate alcohol intake might serve a protective role against HT and other autoimmune conditions, though conclusive evidence is pending [34]. There is no apparent association between HPV infection and the risk of HT. In individuals with HPV infection, HPV vaccination is significantly negatively correlated with the risk of HT.

We further investigated the impact of HPV vaccination on the levels of thyroid autoantibodies. The results of the linear regression analysis revealed a significant negative linear relationship between HPV vaccination and TPOAb/TGAb. The presence and production of thyroid-specific autoantibodies are hallmark characteristics of HT. The majority of individuals with HT generate autoantibodies targeting thyroglobulin (TG) and thyroid peroxidase (TPO), denoted by TGAb and TPOAb, respectively [35]. Moreover, the detection of anti-TPO antibodies is instrumental in anticipating thyroid hypofunction [36]. To date, research investigating the relationship between HPV vaccination and levels of TPOAb/TGAb has been scarce. Our study represents an inaugural effort to examine this association, presenting novel evidence of a significant inverse correlation between HPV vaccination and TPOAb levels. Interestingly, analogous observations have emerged from studies looking at thyroid function post-COVID-19 vaccination. For instance, research by Liubing Li et al. [37]. revealed that individuals with thyroid dysfunction who received the Sinopharm BIBP COVID-19 vaccine experienced reductions in TGAb levels in a significant number of cases (48 out of 77), with all observed abnormal thyrotropin receptor antibody levels diminishing after inoculation. Lower TPOAb levels imply a reduced risk of HT. For individuals with HT, TPOAb is associated with the occurrence of hypothyroidism, and lower TPOAb levels contribute to a better prognosis for patients.

However, our study is not without its constraints. The causal relationships between various factors are not determined due to the limited data available in the NHANES database. The data within NHANES do not extend to clinical features and ultrasound details necessary for a comprehensive diagnosis of HT, confining our study to serum anti-thyroid antibody assessment as the sole diagnostic tool for HT. Furthermore, due to the limited information in NHANES, it is not possible to investigate the randomness of autoimmune diseases and vaccine administration. To summarize, further validation of our findings by rigorously designed prospective studies is needed to provide stronger evidence to support this association.

## 5. Conclusions

The current evidence supports a negative correlation between HPV vaccination and both the occurrence of HT and the levels of TPOAb/TGAb within the studied cohort. These findings suggest a significant association between HPV vaccination and HT, warranting additional causal investigation to validate this inference.

## Figures and Tables

**Table 1:** Baseline characteristics of selected participants from the NHANES 2007–2012.

Characteristic	*n* ^1^	Total, *n* = 39,622,694 ^2^	Unvaccinated *n* = 37,196,826 ^2^	Vaccinated *n* = 2,425,868 ^2^	*p* Value ^3^
Age, years	2717	39 [29, 49]	41 [30, 50]	22 [20, 27]	<0.001
Race	2717				0.027
Non-Hispanic White		1125 (67%)	1053 (67%)	72 (70%)	
Non-Hispanic Black		552 (12%)	518 (12%)	34 (11%)	
Mexican American		511 (8.7%)	496 (9.0%)	15 (3.1%)	
Other Hispanic		341 (6.0%)	313 (5.8%)	28 (9.3%)	
Other		188 (6.5%)	173 (6.5%)	15 (7.2%)	
Education	2561				0.002
Less Than High School		596 (15%)	585 (16%)	11 (4.8%)	
High School		543 (20%)	527 (21%)	16 (12%)	
Some College		829 (34%)	771 (33%)	58 (51%)	
College or above		593 (31%)	564 (31%)	29 (32%)	
BMI status	2693				0.079
underweight		77 (2.8%)	73 (2.9%)	4 (2.3%)	
normal BMI		846 (36%)	775 (36%)	71 (49%)	
overweight		749 (27%)	709 (27%)	40 (24%)	
obese		1021 (34%)	976 (35%)	45 (25%)	
Marital Status	2561				<0.001
Never married		567 (20%)	501 (18%)	66 (53%)	
Married or Living with partner		1518 (64%)	1477 (66%)	41 (40%)	
Divorced or Separated		476 (16%)	469 (17%)	7 (6.8%)	
Alcohol intake	2717				0.9
No		1115 (34%)	1044 (34%)	71 (35%)	
Yes		1602 (66%)	1509 (66%)	93 (65%)	
Smoke	2717				0.013
No		1744 (62%)	1619 (62%)	125 (73%)	
Yes		973 (38%)	934 (38%)	39 (27%)	
High blood pressure	2717				0.005
No		2162 (80%)	2014 (79%)	148 (92%)	
Yes		555 (20%)	539 (21%)	16 (8.2%)	
High cholesterol level	2717				<0.001
No		2147 (77%)	1993 (75%)	154 (96%)	
Yes		570 (23%)	560 (25%)	10 (4.2%)	
Diabetes	2717				0.04
No		2509 (93%)	2350 (93%)	159 (98%)	
Borderline		32 (1.3%)	31 (1.4%)	1 (0.4%)	
Yes		176 (5.5%)	172 (5.7%)	4 (1.9%)	
Ratio of family income to poverty	2516				0.023
high income		910 (51%)	864 (51%)	46 (43%)	
middle income		960 (32%)	908 (32%)	52 (30%)	
low income		646 (17%)	591 (17%)	55 (27%)	
HPV infection	2717				<0.001
No		1492 (59%)	1432 (60%)	60 (37%)	
Yes		1225 (41%)	1121 (40%)	104 (63%)	
TPOAb	2717				0.002
Negative		2374 (86%)	2216 (86%)	158 (97%)	
Positive		343 (14%)	337 (14%)	6 (3.4%)	
TGAb	2717				0.3
Negative		2689 (99%)	2525 (99%)	164 (100%)	
Positive		28 (1.0%)	28 (1.1%)	0 (0%)	
TSH	2716				0.4
0.34–5.6 mIU/mL		2588 (95%)	2430 (95%)	158 (97%)	
<0.34 mIU/mL		71 (2.1%)	66 (2.1%)	5 (2.2%)	
>5.60 mIU/mL		57 (2.6%)	56 (2.7%)	1 (0.8%)	
FT4	2717				0.8
0.60–1.60 ng/dL		2617 (97%)	2458 (97%)	159 (97%)	
<0.60 ng/dL		87 (3.0%)	82 (3.0%)	5 (3.1%)	
>1.60 ng/dL		13 (0.3%)	13 (0.3%)	0 (0%)	

Note: ^1^ *n* not Missing (unweighted); ^2^ *n* (unweighted) (%); Median [IQR]; ^3^ chi-squared test with Rao & Scott’s second-order correction; Wilcoxon rank-sum test for complex survey samples.

**Table 2:** Descriptive characteristics of HPV vaccination.

Girls Vaccinated	164	
Age at vaccination		
18~26	125	76.22%
27~45	34	20.73%
>45	5	3.05%
Number of doses per girl vaccinated *		
1 dose	37	23.87%
2 doses	45	29.03%
3 doses	73	47.10%
Type of vaccine		
2007–2010	Gardasil	
2011–2012	Cervarix/Gardasil	

* 1 Refused, 8 Do not know.

**Table 3:** The association between HPV vaccination and the risk of HT development.

Exposure	Model 1			Model 2			Model 3		
HPV Vaccination	OR	95% CI	*p*-Value	OR	95% CI	*p*-Value	OR	95% CI	*p*-Value
No	-	-		-	-		-	-	
Yes	0.21	0.07, 0.58	0.004	0.28	0.10, 0.78	0.016	0.13	0.02, 0.76	0.025

Note: Model 1: adjusted for none. Model 2: age and race were adjusted. Model 3: age, race, education level, marital status, ratio of family income to poverty, BMI, alcohol intake, diabetes status, smoking, hypertensive state, hyperlipidemia status, and HPV infection were adjusted.

**Table 4:** The association between the number of HPV vaccinations and the risk of HT development.

Exposure	Model 1			Model 2			Model 3		
HPV Vaccination	OR	95% CI	*p*-Value	OR	95% CI	*p*-Value	OR	95% CI	*p*-Value
No	-	-		-	-		-	-	
1 dose	1.05 × 10^−6^	6.56 × 10^−7^, 1.69 × 10^−6^	<0.001	1.63 × 10^−6^	9.87 × 10^−7^, 2.69 × 10^−6^	<0.001	1.72 × 10^−6^	2.02 × 10^−7^, 1.47 × 10^−5^	<0.001
2 doses	0.518	0.129, 2.088	0.3	0.66	0.168, 2.585	0.5	0.247	0.034, 1.796	0.2
3 doses	0.099	0.018, 0.535	0.008	0.136	0.026, 0.725	0.021	9.93 × 10^−7^	1.19 × 10^−7^, 8.32 × 10^−6^	<0.001

Note: Model 1: adjusted for none. Model 2: age and race were adjusted. Model 3: age, race, education level, marital status, ratio of family income to poverty, BMI, alcohol intake, diabetes status, smoking, hypertensive state, hyperlipidemia status, and HPV infection were adjusted.

**Table 5:** The association between HPV vaccination and TPOAb/TGAb.

Exposure	Model 1			Model 2			Model 3		
TPOAb:									
HPV vaccination	Beta	95% CI	*p*-value	Beta	95% CI	*p*-value	Beta	95% CI	*p*-value
No	-	-		-	-		-	-	
Yes	−20.34	−32.11, −8.57	0.001	−14.1	−26.45, −1.74	0.026	−22.27	−34.86, −9.68	0.001
TGAb:									
HPV vaccination	Beta	95% CI	*p*-value	Beta	95% CI	*p*-value	Beta	95% CI	*p*-value
No	-	-		-	-		-	-	
Yes	−6.12	−9.41, −2.84	<0.001	−4.89	−9.63, −0.14	0.044	−7.53	−14.88, −0.18	0.045

Note: Model 1: adjusted for none. Model 2: age and race were adjusted. Model 3: age, race, education level, marital status, ratio of family income to poverty, BMI, alcohol intake, diabetes status, smoking, hypertensive state, hyperlipidemia status, and HPV infection were adjusted.

**Table 6:** The association between the number of HPV vaccinations.

Exposure	Model 1			Model 2			Model 3		
TPOAb:									
HPV vaccination	Beta	95% CI	*p*-value	Beta	95% CI	*p*-value	Beta	95% CI	*p*-value
No	-	-		-	-		-	-	
1 dose	−28.73	−34.19, −23.28	<0.001	−19.91	−26.48, −13.34	<0.001	−22.46	−32.05, −12.88	<0.001
2 doses	−23.59	−32.97, −14.22	<0.001	−17.88	−28.14, −7.62	0.001	−25.37	−43.48, −7.26	0.008
3 doses	−19.6	−38.30, −0.89	0.04	−13.71	−33.08, 5.66	0.2	−26.72	−37.80, −15.64	<0.001
TGAb:									
HPV vaccination	Beta	95% CI	*p*-value	Beta	95% CI	*p*-value	Beta	95% CI	*p*-value
No	-	-		-	-		-	-	
1 dose	−7.26	−10.34, −4.18	<0.001	−5.64	−10.14, −1.14	0.015	−7.7	−14.17, −1.23	0.022
2 doses	−5.76	−9.12, −2.41	0.001	−5.08	−10.47, 0.30	0.064	−6.99	−14.61, 0.64	0.071
3 doses	−5.93	−9.89, −1.97	0.004	−4.53	−9.56, 0.50	0.076	−7.24	−14.36, −0.11	0.047

Note: Model 1: adjusted for none. Model 2: age and race were adjusted. Model 3: age, race, education level, marital status, ratio of family income to poverty, BMI, alcohol intake, diabetes status, smoking, hypertensive state, hyperlipidemia status, and HPV infection were adjusted.

## Data Availability

The datasets analyzed during the current study are available in the National Health and Nutrition Examination Survey (NHANES), https://www.cdc.gov/nchs/nhanes/?CDC_AAref_Val=https://www.cdc.gov/nchs/nhanes/index.htm (accessed on date 31 January 2025).

## Abbreviations

The following abbreviations are used in this manuscript:

BMI	body mass index
TGAb	thyroglobulin antibodies
TPOAb	thyroid peroxidase antibodies
TSH	thyroid-stimulating hormone
FT4	free thyroxine
OR	odds ratio
CI	confidence interval
95% CI	95% confidence interval

## Appendix A

**Table A1:** The association between HPV infection and Hashimoto’s thyroiditis.

Exposure	Model 1			Model 2			Model 3		
HPV Infection	OR	95% CI	*p*-Value	OR	95% CI	*p*-Value	OR	95% CI	*p*-Value
No	-	-		-	-		-	-	
Yes	0.78	0.58, 1.05	0.1	0.92	0.67, 1.26	0.6	1.03	0.72, 1.48	0.9

Note: Model 1: adjusted for none. Model 2: age and race were adjusted. Model 3: age, race/ethnicity, education level, marital status, ratio of family income to poverty, BMI, alcohol intake, diabetes status, smoking, hypertensive state, hyperlipidemia status, and HPV infection were adjusted.
